# Rehmanniae Radix Preparata ameliorates behavioral deficits and hippocampal neurodevelopmental abnormalities in ADHD rat model

**DOI:** 10.3389/fnins.2024.1402056

**Published:** 2024-05-30

**Authors:** Ruxin Sun, Haixia Yuan, Jing Wang, Kanglin Zhu, Yu Xiong, Yabei Zheng, Xinqiang Ni, Min Huang

**Affiliations:** ^1^Department of Neurology, The Seventh Affiliated Hospital of Sun Yat-sen University, Shenzhen, China; ^2^Affiliated Hospital of Nanjing University of Chinese Medicine, Nanjing, China; ^3^The Fourth Clinical Medical College, Guangzhou University of Chinese Medicine, Shenzhen, China

**Keywords:** ADHD, Radix Rehmanniae Preparata, spontaneously hypertensive rats, hippocampus, neurodevelopment

## Abstract

**Objectives:**

Abnormal hippocampal neurodevelopment, particularly in the dentate gyrus region, may be a key mechanism of attention-deficit/hyperactivity disorder (ADHD). In this study, we investigate the effect of the most commonly used Chinese herb for the treatment of ADHD, Rehmanniae Radix Preparata (RRP), on behavior and hippocampal neurodevelopment in spontaneously hypertensive rats (SHR).

**Methods:**

Behavior tests, including Morris water maze (MWM) test, open field test (OFT) and elevated plus maze (EPM) test were performed to assess the effect of RRP on hyperactive and impulsive behavior. Hippocampal neurodevelopment was characterized by transmission electron microscopy, immunofluorescence, Golgi staining and Nissl staining approaches. Regulatory proteins such as Trkb, CDK5, FGF2/FGFR1 were examined by Western blot analysis.

**Results:**

The results showed that RRP could effectively control the impulsive and spontaneous behavior and improve the spatial learning and memory ability. RRP significantly reduced neuronal loss and increased the number of hippocampal stem cells, and promoted synaptic plasticity. In addition, FGF/FGFR signaling was upregulated after RRP treatment.

**Conclusion:**

RRP can effectively reduce impulsive and spontaneous behavior and ameliorate hippocampal neurodevelopmental abnormalities in ADHD rat model.

## 1 Introduction

Attention-deficit/hyperactivity disorder (ADHD) is widely recognized as the most common neurodevelopmental disorder in children characterized by hyperactivity, inattention and/or impulsivity (Contreras et al., 2022), with a prevalence of over 5% (Thomas et al., 2015). The etiology of ADHD is related to a variety of factors, and the pathogenesis of ADHD has not been clarified (Bonvicini et al., 2016). Neuroimaging has revealed that cortical surface area and several subcortical structures, including the hippocampus, are reduced in size in ADHD patients (Plessen et al., 2006; Perlov et al., 2008; Hoogman et al., 2017). Interestingly, the hippocampus is the brain region that shows the most significant reduction and delayed volume maturation in childhood (Hoogman et al., 2017). Thus, a neurodevelopmental disorder of the brain has been proposed as a cause of ADHD (Shaw et al., 2007). Adult hippocampal neurogenesis plays a key role in hippocampal plasticity and its dysfunction is implicated in cognitive disorders (Wang Y. et al., 2021). Neurogenesis occurs throughout the mammalian lifespan, mainly in the dentate gyrus (DG) of the hippocampus (Ming and Song, 2011; Song et al., 2016). In this region, neural stem cells (NSCs) constantly proliferate, leading to the generation of new neurons (Bergmann et al., 2015). A hallmark of neural development is the maturation of dendritic spines. Dendritic spines exhibit changes in density, morphology and function during neural development and remodeling (Maiti et al., 2015). In addition, several psychostimulants such as methylphenidate (MPH) have been shown to reduce hyperactivity by restoring neuroplasticity in ADHD model rats (1). In summary, hippocampal neurodevelopment, particularly in the DG region, may be a key mechanism in the pathogenesis of ADHD. Furthermore, previous studies have shown that several genes, such as Tyrosine kinase receptor B (Trkb) (Liu et al., 2015), Cyclin-dependent kinase 5 (Cdk5) (Cortés et al., 2019), fibroblast growth factor/fibroblast growth factor receptor (FGF/FGFR) (Mooney et al., 2016), are associated with adult neurogenesis in the hippocampus, which may help to investigate the underlying molecular mechanisms of ADHD.

The most widely used medications for ADHD are psychostimulants, such as methylphenidate (MPH) and tomoxetine hydrochloride. Approximately 30% of the children with ADHD cannot tolerate the side effects of psychostimulants (appetite loss, sleep disturbance, drug dependence, etc.) (Goez et al., 2007; Groenman et al., 2017; McCarthy et al., 2018). The existence of the above factors makes it the search for multi-component, multi-safer and more effective drugs from traditional Chinese medicine for ADHD in recent years. Radix Rehmanniae Preparata (RRP) was extracted from the root tuber of the plant Rehmannia glutinosa. According to modern research and clinical studies, RRP and its active components are of great neuroprotective effects and have been widely used in Parkinson’s disease (PD), Alzheimer’s disease (AD), stroke by alleviating energy metabolism failure and preventing neuronal apoptosis. These characteristics are related to the pathogenesis of ADHD (Jia et al., 2023). According to a data mining report Radix Rehmanniae Preparata (RRP) was the most commonly used herbal medicine for ADHD (Ni et al., 2015). Recent evidence revealed that RRP administration could reduce spontaneous and impulsive behaviors in young spontaneously hypertensive rats (SHR) by upregulating BDNF/TrkB and NRG-3 expression, and improve their learning and memory abilities (Yuan et al., 2018).

Spontaneously hypertensive rats (SHRs) are considered as the gold standard model rats for ADHD in basic research (Sagvolden, 2000), because SHR have impulsiveness, increased motor activity and cognitive impairment (Tchekalarova et al., 2023). And they are often compared to rats from the same ancestral inbred Wistar colony, the Wistar Kyoto rat (WKY). Similar to children with ADHD, SHR have been found to have reduced brain volume (Sagvolden et al., 2005). SHR also show neurochemical impairments such as cortical striatal dopaminergic hypoplasia (de Santana et al., 2020).

In the present study, we tested the hypothesis that RRP could ameliorate ADHD-like behaviors and improve hippocampal neurodevelopment by inhibiting apoptosis and enhancing neurogenesis and synaptic plasticity using SHR model rats. This is the first study to address the role of impaired hippocampal neurodevelopment and plasticity in the pathophysiology of ADHD and to search for alternative treatments of such disorder.

## 2 Materials and methods

### 2.1 Animals

Thirty SPF-grade SHR rats and 10 WKY rats, all males, 3 weeks old, were provided by Beijing Vital River Laboratory Animal Technology Co., Ltd. (Beijing, China, Certificate No. SCXK (Jing)2021-0006). All animals were housed individually at a controlled temperature of 20–23°C with a controlled light exposure of 12 h light/12 h dark cycle at 300 lux. Animal experiment was approved by the Laboratory Animal Ethics Committee of The Second People’s Hospital of Shenzhen. All rats were given free access to water and standardized rat food. Animals were acclimatized and fed for 5 days prior to behavioral testing and gavage administration.

### 2.2 Preparation of Rehmanniae Radix Preparata extract

RRP was purchased from Beijing TongRenTang pharmacy (Beijing, China). For aqueous extraction of RRP, dried RRP was cut into small pieces and reflux-extracted with distilled water at 60°C three times. The extract of RRP was then concentrated using a vacuum evaporator. The supernatants were harvested and dried the water in a vacuum freeze-dryer (−80°C). Finally, the extracts were combined and stored at −20°C until use.

We used UHPLC–MS analysis to analyze the composition of RRP. UHPLC–MS analysis was performed on an Acquity UPLC system and Waters Xevo G2 Q-Tof mass spectrometer. Chromatographic separations were performed on Waters Acauity UPLC HSS T3 column (100 mm × 2.1 mm, 1.8 μm, Waters, Milford, MA, United States) at 25°C, and the injection volume was 5 μL at a flow rate of 0.4 mL/min.

### 2.3 Administration of RRP and MPH

After 7 days of acclimatization, the open field test (OFT) was performed to observe the spontaneous behavior of the rats (4 weeks of age). The total distance of movement was used as an indicator, and random block design was performed on SHR rats using Excel software. SHR were divided into three groups (*n* = 10 in each group): SHR group, RRP group, and MPH group. The administered dose of RRP was converted between rat and human clinical doses by referring to the methodology of “Methodology of Pharmacological Research of Traditional Chinese Medicines,” and the final concentration in rats by gavage was calculated to be 0.12 g/mL. Combined with the literature and the above methodology, the concentration in the MPH group was 0.1 mg/mL.

**Figure 1:**
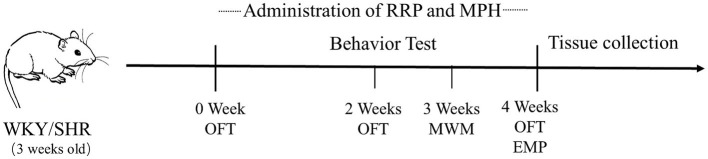
Schedule of the experimental timing.

RRP (2.4 g∙kg) and MPH (2 mg∙kg) were dissolved in 0.5% CMC-Na before administration. Both the WKY and SHR groups were given 0.5% CMC Na solution at 2 mL/(kg∙d) by gavage. After daily weighing, the drug was administered by gavage twice at 9:30 and 15:30 for 4 weeks. Behavioral tests were performed on rats during the night cycle (9,00–21,00), 30 min after the first daily dose and before the second dose. Rats underwent all of the following behavioral tests: elevated plus maze (EPM; week 4), open field test (OFT; weeks 0, 2, and 4), and Morris water maze (MWM) (week 3). The day after the behavioral experiments, brain tissue was dissected for experimental purposes or stored at −80°C for further analysis. The time schedule was provided in Figure 1.

### 2.4 Open field test (OFT)

The open field test was used to assess the spontaneous activity and impulsive behavior of the animals (Liu et al., 2023), and was conducted before the administration of the drug, 2 weeks after the administration of the drug, and 4 weeks after the administration of the drug (at 4, 6 and 8 weeks of age in rats). The bottom of the opening chamber was divided into 16 squares of 25 cm × 25 cm using the animal behavioral activity recording and analysis system. The rats were placed in the central area of the experimental box, and their total movement distance (m), average speed (cm/s), number of entries into the central area (the middle four squares), and movement distance (m) in the central area were automatically recorded by the Easy-Tracking System (SLY-ETS Version 1.66, Beijing Sunny Instruments Co. Ltd.) within 5 min. At the end of each rat test, the feces were cleaned up and the experimental chamber was sprayed with 75% alcohol (*N* = 10 rats/group).

### 2.5 Morris water maze (MWM)

The Morris water maze was performed at 3 weeks of treatment (at 7 weeks of age in rats). The experiment was conducted for 6 consecutive days, with hidden station experiments on days 1 to 5 and a spatial exploration experiment on day 6, with a 24 h interval between each behavioral test. The analysis system divided the maze into four quadrants, and the platform (12 cm in diameter and 30 cm in height) was placed in the center of a quadrant, with the water surface in the pool approximately 1 cm above the platform. The water temperature was maintained at (24 ± 1)°C. The hidden station experiment was conducted four times a day. One quadrant was selected randomly each time, and the rats swam for a limited time of 90s. If the rat could climb on the platform within the specified time and stay on the platform for more than 10s, it was considered that the rat had found the platform. During the spatial exploration experiment, the platform was withdrawn, and the time required for the rats to enter the original platform area (latency) (s), the number of times the rats crossed the platform within 90 s, and the residence time in the target quadrant (the quadrant where the platform was located before it was withdrawn) (s) were recorded by the Easy-Tracking System (SLY-ETS Version 1.66, Beijing Sunny Instruments Co. Ltd.). The maze was surrounded by a black color blackout cloth to avoid interference from the external environment (*N* = 10 rats/group).

### 2.6 Elevated plus maze (EPM) test

EPM test was performed at the end of the opening experiment after 4 weeks of administration (at 8 weeks of age in rats). The analysis system divided the elevated cross maze into open arm, closed arm and central area. During the test, rats were placed in the central area of the elevated cross maze with their heads facing the open arm, and the total movement distance (m), the ratio of the number of entries into the open arm and the residence time (s), and the residence time in the central area of the maze were recorded by the Easy-Tracking System (SLY-ETS Version 1.66, Beijing Sunny Instruments Co. Ltd.) within 5 min (*N* = 10 rats/group).

### 2.7 Sample collection

After behavioral testing, the rats (*N* = 10 per group) were fasted overnight and anesthetized with isoflurane (RWD, R510-22-10). For brain tissue staining, four rats in each group were randomly selected. Rats were injected with 100 mM cold PBS (pH 7.4), followed by 4% cold paraformaldehyde solution (PFA) until fixed convulsions were observed in the extremities. The brains were immediately isolated and fixed in 4%PFA at 4°C for 24 h. The brains embedded in paraffin, and cut on a standard microtome (Leica, Wetzlar, Germany) into slices with a thickness of 5 μm. Different methods of brain tissue staining, including immunofluorescence, TUNEL staining and Nissl staining, were used to analyze the brain tissue slices of each group. For the remaining rats in each group, rats were given a cardiac infusion of cold phosphate buffered saline (PBS). The entire brain was removed from the skull and then the left and right hippocampus were quickly dissected on ice. For the analysis of TEM, the total hippocampus in the left hemisphere of each rat was dissected into 1 mm^3^ and fixed (*N* = 3 rats/group). The right hippocampus of each rat was immersed in Golgi-Cox staining solution (*N* = 3 rats/group). For western blot analysis, the hippocampus was quickly dissected on a cold dissecting table after perfusion, weighed and frozen in liquid nitrogen (*N* = 3 rats/group).

### 2.8 Transmission electron microscopy

The hippocampus tissues were removed and rinsed in 0.1 M PBS three times. Tissues were fixed in 1% osmium tetroxide in 0.1 M PBS for 2 h and rinsed three times with PBS. Tissues were dehydrated sequentially in a gradient of 50–70%-80–90%-95–100-100% alcohol-100% acetone-100% acetone for 15 min each time, and then impregnated with a mixture of 50% epoxy resin and 50% acetone for 2–4 h at room temperature. The sample inserted into the embedding plate after 37°C oven overnight, then incubated at 60°C for 48 h to polymerize. 2.5% aqueous uranyl acetate and Reynold’s lead citrate double staining, each stained for 15 min. The sections were dried at room temperature overnight. Transmission electron microscopy was used for observation, images were collected and analyzed (*N* = 3 rats/group).

### 2.9 Nissl staining

The hippocampus tissues were placed in 4% paraformaldehyde (Servicebio, cat: G1101) for 24 h and then embedded in paraffin. Cut paraffin to 5 μm thickness for Nissl staining. The slices were baked at 60°C for 2 h on a spreading baking machine, to prevent the slices from falling off. The brain slices were dewaxed, rehydrated, and submerged in 0.1% cresyl violet staining solution for 12 min (37°C). The slices were submerged in 70% alcohol (AC) for 30 s, 80% AC for 30 s, 95% AC I for 30 s, 95% AC II for 30 s, AC I for 5 s, AC II for 5 s. After drying the slices, the slices were submerged in Dibenzoylmethane (DMB) I for 2 min, and DMB II for 2 min, and finally the slices were sealed by fixing them with neutral balsam. The quantification of Nissl bodies in the hippocampal CA1, CA3, and DG regions was conducted using Image J software. A total of three slices were analyzed for each group (*N* = 3 rats/group).

### 2.10 Golgi-Cox staining and Sholl analysis

Golgi Cox staining refers to neuronal staining used to assess synaptic plasticity. Fresh brain tissue was extracted from 3 rats in each group and washed with PBS. The brain tissue was completely immersed in Golgi-Cox staining solution (Servicebio, Wuhan, China) and placed in a cool place away from light for 14 days. The tissue blocks were then removed for dehydration and treated with a developer for 45 min. Finally, the sample was dehydrated, frozen, sliced and sealed with glycerin gelatin. Images of the hippocampal neurons and dendritic spine in CA1, CA3 and DG regions were taken by Upright electron microscope (NIKON, JAPAN) with CaseViewer software. And the images were analyzed using ImageJ software and GraphPad Prism 8.0. For each group (*N* = 3 rats/group), a total of three sections were analyzed.

The effect of RRP on dendritic complexity was investigated by Sholl analysis. Transparent concentric spherical grids called the Sholl ring were created using Fiji software. Dendritic branches beginning from cell soma, and then we counted various morphometric parameters in each successive concentric ring at 20 μm intervals, such as number of intersections (*N* = 3 rat/group).

### 2.11 TUNEL staining

We used the dUTP-nick-end labeling (TUNEL) apoptosis assay kit (Beyotime, C1086) for TUNEL staining. The brain slices were dewaxed, rehydrated in decreasing concentrations of ethanol. The sample was treated with 1%Triton X-100 and permeated at room temperature for 3-5 min. 100 μL proteinase K was added to each sample at 37°C for 30 min, then PBS washed three times. Prepared TUNEL test solution, then we added TUNEL test solution to cover the sample. Fluorescence microscope was observed after sealing with anti-fluorescence quenching sealing solution. The percentage of TUNEL cells out of total cells (DAPI) were detected in the DG area indicating apoptosis in SHR. For each group (*N* = 3 rats/group), a total of three sections were analyzed.

### 2.12 Immunofluorescence staining

Paraffin sections were deparaffinized in xylene and rehydrated through graded ethanol solutions. Two drops of 3% H2O2-methanol solution were added to each section and treated at room temperature for 10 min. Then, 50–100 μL of goat serum was added and incubated at room temperature for 20 min. After removing blocking buffer, cells were incubated with primary antibodies overnight at 4°C. We used rabbit anti-NeuN (1:100) (CST, #24307) for degree of neuronal developmental maturation, anti-Nestin (1:500) (Servicebio, GB12137) for neural stem cells and mouse anti-Ki67 (1:200) (Servicebio, cat: GB111499) for cell proliferation. Secondary antibodies were Alexa Fluor 488 goat anti-rabbit antibodies (1:500) (Servicebio, GB25303) and Cy3-AffiniPure goat anti-mouse antibodies (1:500) (Servicebio, GB21301). All samples were counterstained with DAPI dye to label cell nuclei. The number of Ki67 and Nestin double-labeled positive cells in the DG region was counted using ImageJ software. The percentage of NeuN positive cells out of total cells (DAPI) were quantified in the DG area indicating mature neurons. A total of three slices were analyzed for each group (*N* = 3 rats/group).

### 2.13 Western blot analysis

The isolated hippocampus was homogenized with a tissue grinder and then lysed on ice for 30 min in cold RIPA buffer containing PMSF (Beyotime Biotechnology, China). Ep tubes centrifuged at 4°C,12000 rpm for 10 min. The supernatant was taken up as total protein. Protein concentration in the supernatant was measured by BCA protein assay kit (Beyotime, cat: P0012). Protein samples were mixed with the loading buffer, and boiled at 100°C for 5 min. Subsequently, protein was loaded in each lane, separated using 10 or 12% polyacrylamide gels separated by SDS–PAGE and then transferred to PVDF membrane. Then membranes were blocked in 5% skim milk powder at room temperature and blotted overnight at 4°C with primary antibodies: antibodies to Trkb (Abcam: ab187041, 1:5000), FGF21 (Abcam: ab171941, 1:1000), Wnt3a (Abcam: ab219412, 1:1000), β- actin (Santa Cruz:sc-47778, 1:10000). The membranes were then incubated with corresponding secondary antibody: Goat anti-Rabbit IgG (HRP) (Abcam: ab6721, 1:10000) for 1 h at room temperature. The immunoblot bands were developed using enhanced chemiluminescence (ECL) solution, then captured and analyzed with ChemiDoc MP Imaging System (Bio-Rad, United Stataes) (*N* = 3 rats/group).

### 2.14 Image analysis and statistical analysis

Statistical analysis was conducted using GraphPad Prism 8.0.2. Image J-Fiji software was used to analyze the optical density in western blot and the number of positive cells in the fluorescence images. The data are expressed as the mean ± standard deviation (SD). All data were tested for normal distribution with the Normality and Lognormality test and Brown–Forsythe test for equal variances. One-way analysis of variance (ANOVA) or two-way ANOVA was followed by Tukey’s *post hoc* test. Differences were considered significant at *p*-value < 0.05.

## 3 Results

### 3.1 UHPLC–MS analysis of RRP extract

**Figure 2:**
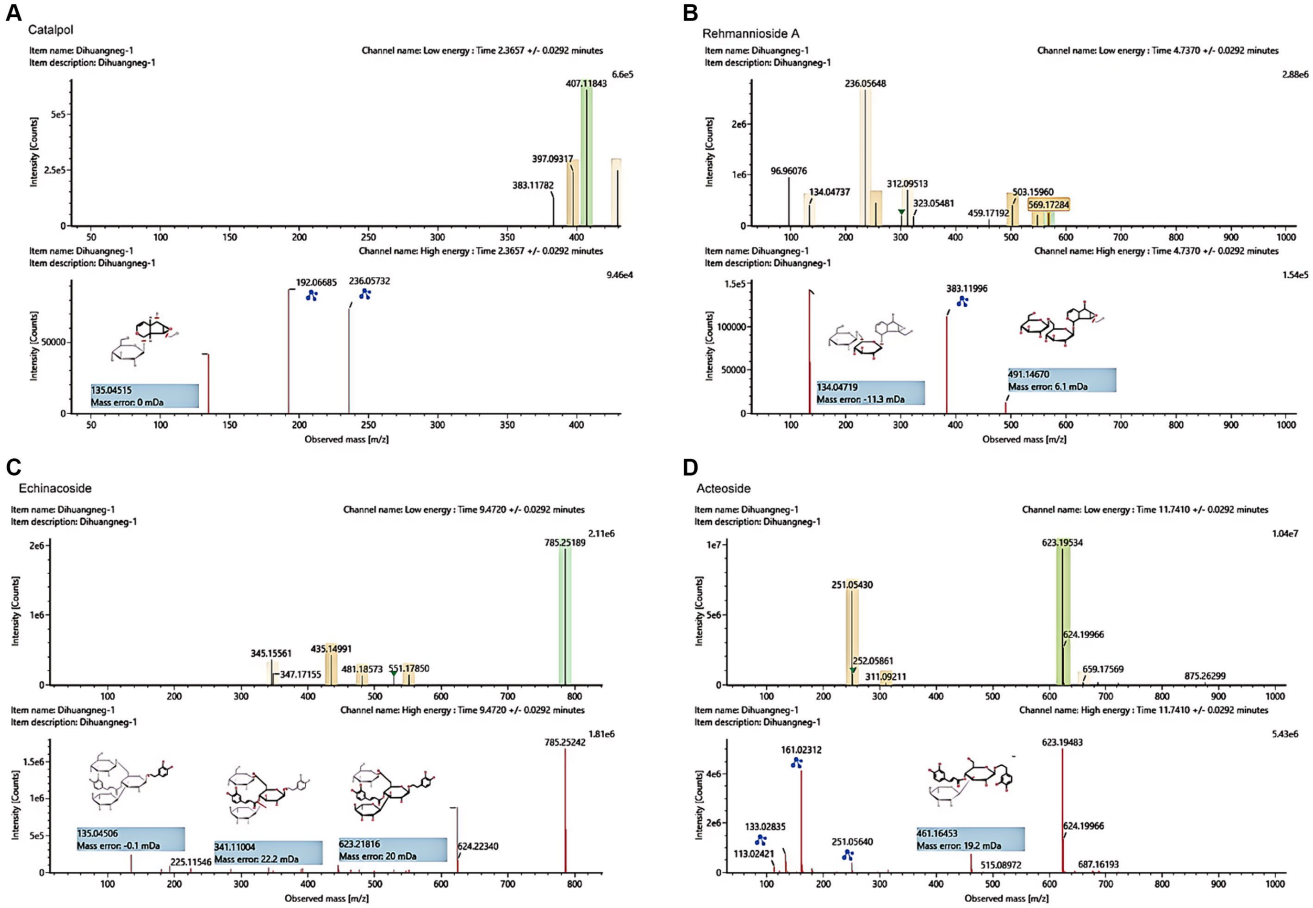
UHPLC–MS characteristics of RRP extract. **(A)** Catalpol in the RR extract sample. **(B)** Rehmannioside A in the RRP extract sample. **(C)** Echinacoside in the RRP extract sample. **(D)** Acteoside in the RRP extract sample.

The UHPLC–MS characteristics of RRP extract were detected. As shown in Figure 2, we successfully identified several compounds, including catalpol, rehmanniosides, acteoside, and echinacoside.

### 3.2 Effect of RRP on impulsivity and hyperactivity in SHR

**Figure 3:**
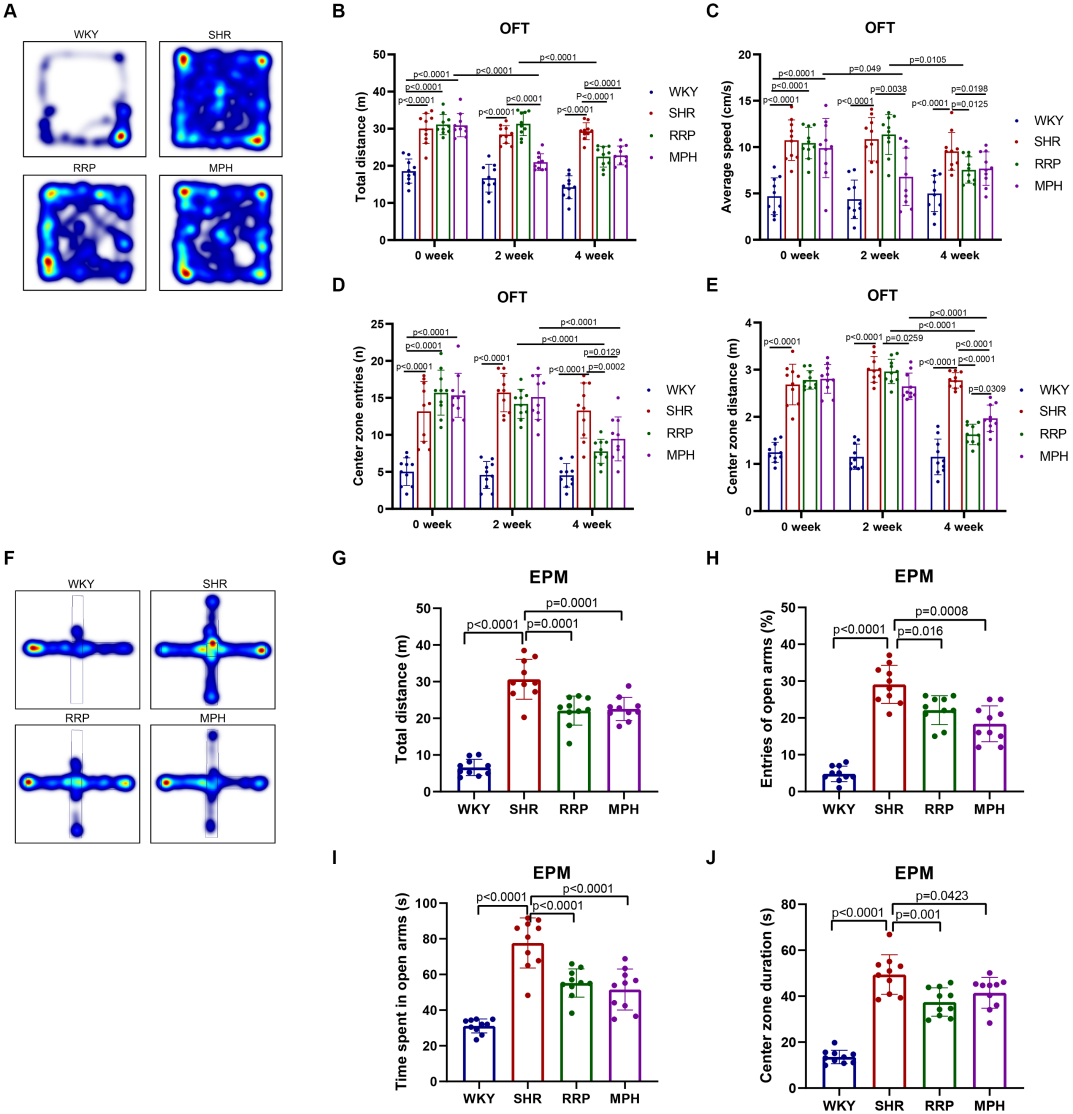
Behavioral performance in OFT and EPM test. **(A,F)** Representative track map of OFT and EPM test from different groups. In the open field test, the above figure showed the results of **(B)** total distance traveled, **(C)** average speed, **(D)** center zone entries and **(E)** center zone distance traveled. In the elevated plus-maze test, the figure showed the results of **(G)** total distance, **(H)** percentage open arm entries, **(I)** open arm duration, **(J)** center duration. All data are expressed as the mean ± SD. *N* = 10 rats/group; one-way ANOVA followed by Tukey multiple test.

The elevated plus maze (EPM) test and the open field test (OFT) are used to investigate anxiety-like, spontaneous behavior in rats (Canseco-Alba et al., 2021; Kohe et al., 2023). The OFT is a classic experiment to evaluate the spontaneous behavior of experimental animals, and has been widely used in neurological and psychopharmacological studies (Walsh and Cummins, 1976). The results of OFT showed that compared with the WKY group, the total distance (Figure 3B, *F*(3, 36) = 33.52, *p* < 0.0001), average speed (Figure 3C, *F* (3, 36) = 14.98, *p* < 0.0001), center zone entries (Figure 3D, *F* (3, 36) = 26.18, *p* < 0.0001) and center zone distance (Figure 3E, *F* (3, 36) = 63.76, *p* < 0.0001) of the other three groups that SHR, MPH, RRP groups before drug administration were all significantly increased (*p* < 0.05). At 2 weeks of drug administration, compared with the SHR group, the total distance (*F* (3, 36) = 51.74, *p* < 0.0001), average speed (*F* (3, 36) = 18.63, *p* < 0.0001) and center zone distance (*F* (3, 36) = 104.3, *p* < 0.0001) were significantly lower in the MPH group (*p* < 0.05), but RRP groups had no significant change. After 4 weeks of drug administration, the levels of all indicators in the MPH and RRP groups decreased significantly compared with those in the SHR group, and the differences were statistically significant (the total distance: *F* (3, 36) = 52.91, *p* < 0.0001; average speed: *F* (3, 36) = 10.55, *p* < 0.0001; center zone entries: *F* (3, 36) = 19.11, *p* < 0.0001; center zone distance: *F* (3, 36) = 62.97, *p* < 0.0001). And the center zone distance of RRP group rats was significantly shorter than MPH group (*p* < 0.05).

EPM test was designed based on the conflict between the curiosity of mice facing new things (open arm) and their darkness-loving nature (closed arm) (Walf and Frye, 2007). In EPM test, SHR, MPH and RRP groups had longer distance (Figure 3G, *F* (3, 36) = 67.2, p < 0.0001), more open arm entries (Figure 3H, *F* (3, 36) = 59.45, *p* < 0.0001), time spent in the open arms (Figure 3I, *F* (3, 36) = 35.75, *p* < 0.0001) and central area (Figure 3J, *F* (3, 36) = 57.63, *p* < 0.0001) than WKY rats. And we found that MPH and RRP treatment could reduce the number of entries into the open arm and the duration in the open arm. In addition, MPH and RRP significantly reduced the total exercise distance of SHR rats. These confirmed the effectiveness of MPH and RRP in reducing the spontaneous behavior of SHR rats.

### 3.3 RRP improved spatial learning memory in SHR

**Figure 4:**
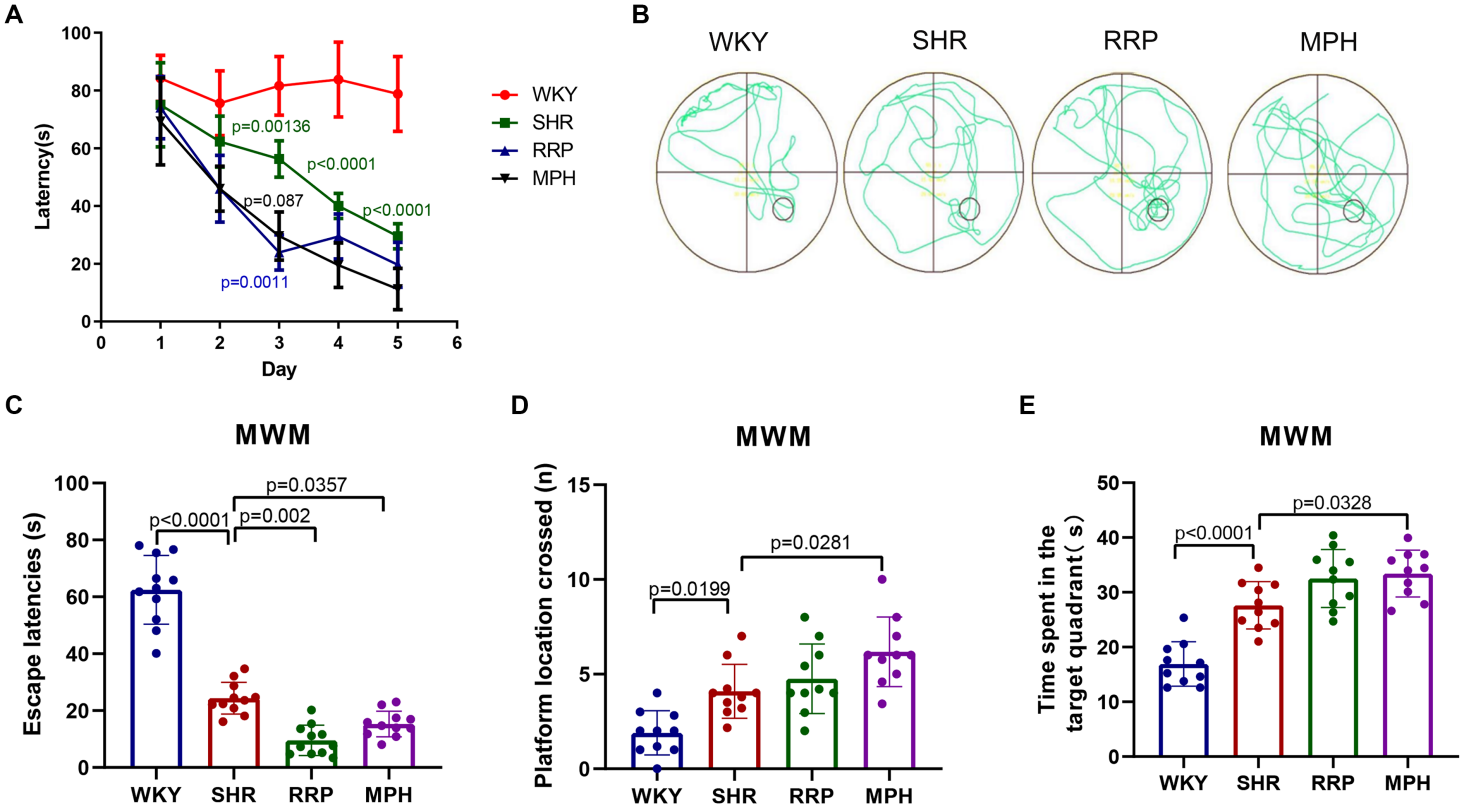
Effect of RRP on activity of SHR rats in MWM test. **(A)** Line chart of latency in hidden platform test of SHR rats in MWM. In day 3, *p* = 0.00136, SHR versus WKY group; *p* = 0.0011, *p* = 0.0087, compared to SHR group. In day 4 and 5, *p* < 0.0001, SHR versus WKY group. **(B)** Representative track map of the Morris water maze test from different groups. **(C)** Escape latency (s): time taken by rats to enter the original platform area. **(D)** The number of platform location crossed in exploratory experiment. **(E)** Time spent in the target (south-east) quadrant (s) in exploratory experiment. All data are expressed as the mean ± SD. *N* = 10 rats/group; one/two-way ANOVA followed by Tukey multiple test.

The study indicates that children and adolescents with ADHD have learning and memory difficulties (Andersen et al., 2013). The Morris water maze experiment has become a classic experiment for studying and evaluating the spatial learning and memory abilities of animals, and can be used to study the effect of RRP on the learning and memory abilities of SHR (Valencia-Olvera et al., 2023). The results of the MMW hidden platform test showed that the latency of the other groups was significantly decreased compared with the WKY group at the same time (*p* < 0.05). Compared with the same time SHR group, the latency period of MPH group and RRP group was significantly decreased on day 3 (*p* < 0.05) (Figure 4A, Two-way ANOVA indicated a significant effect of day [*F* (4, 32) = 30.15, *p* < 0.0001], treatment conditions [*F* (3, 8) = 81.20, *p* < 0.0001] and interaction [*F* (12, 32) = 3.933, *p* = 0.001]. To assess memory retention, an exploratory experiment was conducted without the platform 24 h after the last training session (Figures 4C–E). The results of the MWM exploratory experiment showed a significant decrease in escape latency (*p* < 0.05) and a significant increase in the number of crossing platforms and residence time in the target quadrant (*p* < 0.05) in all other groups compared to the WKY group. Compared with the SHR group, the escape latencies of MPH group and RRP group decreased significantly (Figure 4C, *F* (3, 36) = 110.4, *p* < 0.0001). However, compared with the SHR group, the number of crossing platforms (Figure 4D, *F* (3, 36) = 12.55, *p* < 0.0001) and the residence time in the target quadrant (Figure 4E, *F* (3, 36) = 12.55, *p* < 0.0001) increased significantly in MPH group, not RRP group.

### 3.4 Effect of RRP on ultrastructure and survival of hippocampal neurons

**Figure 5:**
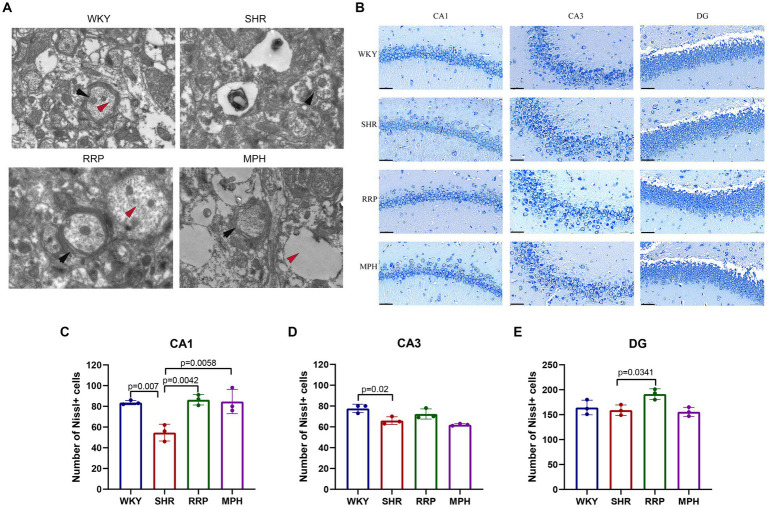
Transmission electron microscopy and Nissl’s staining in the hippocampus. **(A)** Representative image of TEM. The black arrow pointed to the myelin sheath and the red arrow pointed to the microtubule and microfilament structure within the axon. **(B)** Nissl-stained neurons in the CA1, CA3 and DG region of the hippocampus. Scale bar = 50 μm. **(C–E)** Quantified Nissl’s staining analysis results. All data are expressed as the mean ± SD. *N* = 3/group; one-way ANOVA followed by Tukey multiple test.

Transmission electron microscopy (TEM) is often used to observe the ultrastructure of tissues, and it has been preliminarily applied to observe the ultrastructure of the brain neurons of WKY and SHR rats. As shown in Figure 5A, the myelin sheath of neurons in the hippocampus of the WKY group was relatively intact and dense, and the microfilament microtubule structure could be seen in the axon. The myelin sheath of neurons in the SHR group was loose, and the nerve fiber layer was swollen and broken in different degrees. Compared with the WKY rats, the structure of many microfilaments and microtubules in axons disappeared. In the myelin sheath of the MPH group, the nerve fiber layer of the myelin sheath was disrupted and the microfilament microtubule structure disappeared. However, the nerve fiber layer of the myelin sheath of the RRP group could be seen to be disrupted but its structure was still dense, and its detachment was reduced compared to that of the SHR group, and the disappearance of the microfilament microtubule structure was not evident in the axon.

Nissl body is a characteristic structure of the nucleus of neurons (Kádár et al., 2009). When neurons are stimulated, the Nissl bodies in the cell are significantly reduced or even disappear. Therefore, the presence and disappearance of Nissl bodies can reflect whether neurons are damaged. In this manuscript, the number of surviving Nissl bodies was calculated to determine the survival of neurons using ImageJ software. As presented in Figure 5C, the number of surviving neurons stained by Nissl’s presented a significantly decreased change in CA1 and CA3 area of the SHR group (*p* < 0.05). Besides, RRP and MPH treatment increased the number of Nissl bodies in CA1 area (Figure 5C, *F* (3, 8) = 11.86, *p* < 0.0026). However, there was no significant difference in the number of Nissl bodies in CA3 area of SHR group and drug administration group (Figure 5D, *F* (3, 8) = 10.48, *p* < 0.0038). The number of Nissl bodies in DG area of SHR group and RRP group was significantly different (Figure 5E, *F* (3, 8) = 6.091, *p* < 0.0184). The results indicated that administration of RRP treatment contributed to the survival of neuronal cells in SHR rats.

### 3.5 RRP reduced apoptosis in SHR rats

**Figure 6:**
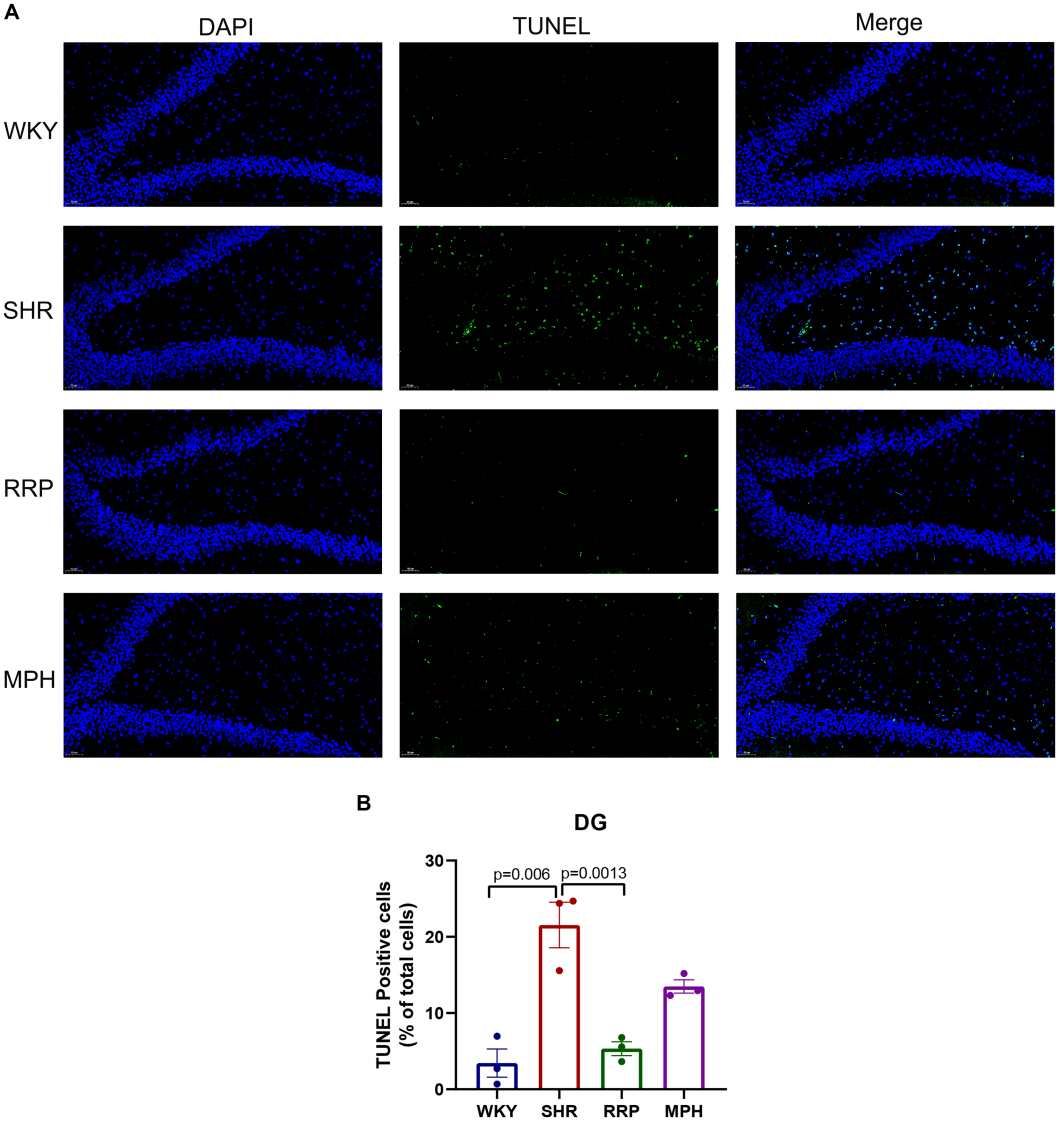
Apoptosis of dentate gyrus. **(A)** TUNEL staining plots of hippocampal apoptosis in each group of rats, TUNEL-positive cells were stained green; Scale bar = 50 μm. **(B)** Relative percentage of TUNEL—positive cells in the hippocampal DG region in each group of rats. All data are expressed as the mean ± SD. *N* = 3/group; one-way ANOVA followed by Tukey multiple test.

We used TUNEL staining to observe the apoptosis in the dentate gyrus of rats (Figure 6). The increase in the number of TUNEL-positive cells demonstrated a significant increase in apoptosis in the hippocampal DG area of SHR rats compared with WKY rats (*p* = 0.006). And the decrease in the number of TUNEL-positive cells in the DG area after RRP treatments suggested that the inhibitory effects of RRP on apoptosis in DG (Figure 6B, *F* (3, 8) = 19.69, *p* < 0.0005).

### 3.6 RRP enhanced synaptic plasticity in hippocampal DG

**Figure 7:**
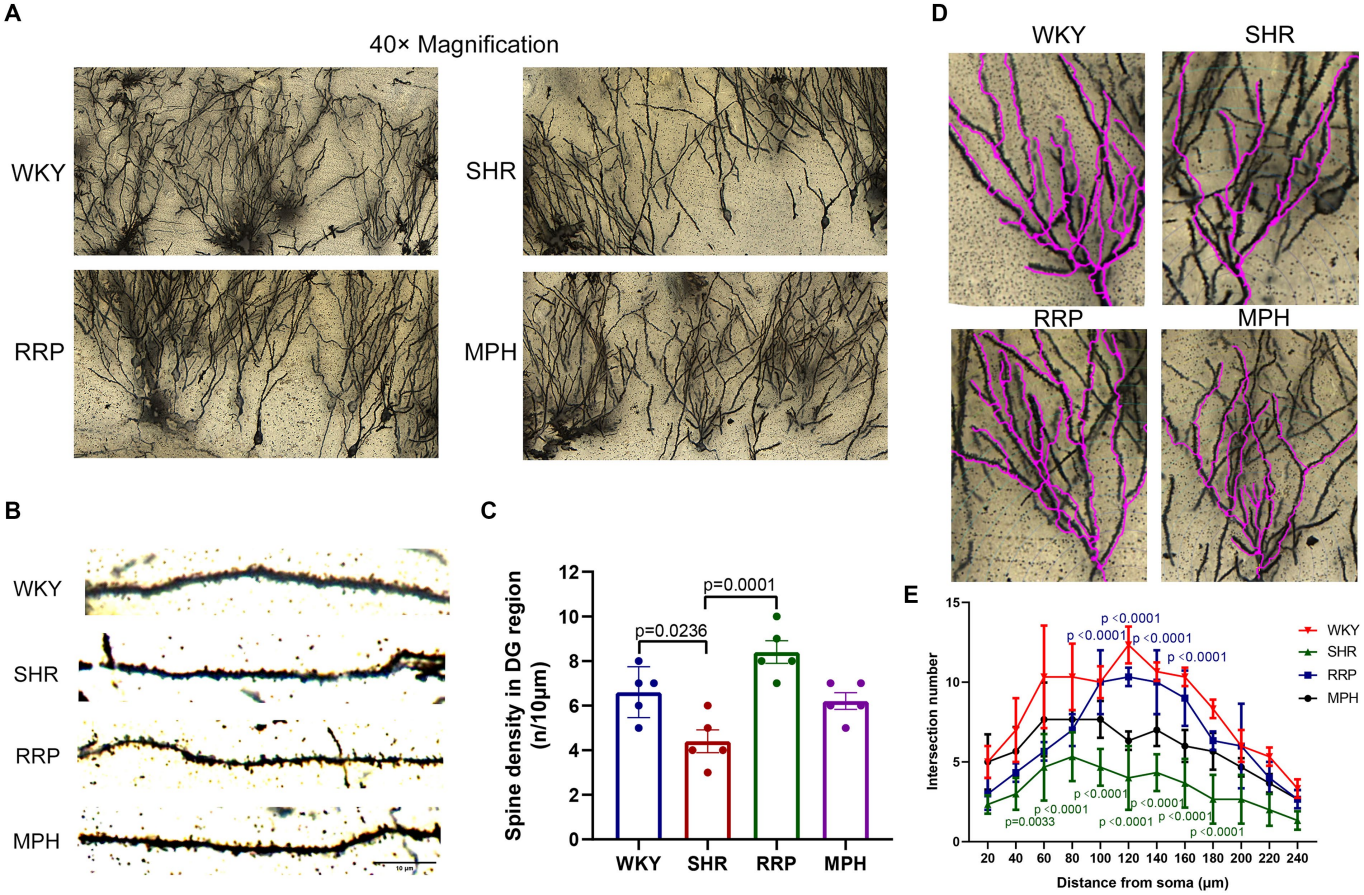
Golgi staining of hippocampus. **(A)** Golgi staining of hippocampal DG neurons in each group. **(B)** Golgi staining of dendritic spines in hippocampal DG neurons from each group. Scale bar = 10 μm. **(C)** Corresponding quantitation of dendritic segments of DG neurons in each group. **(D,E)** Sholl analysis of the number of dendritic branches with concentric circles in hippocampal DG neurons in each group. In the distance of 60–180 μm, *p* = 0.0033, SHR versus WKY group. In the distance of 40 μm, *p* < 0.0001, SHR versus WKY group. In the distance of 60–180 μm, *p* < 0.0001, RRP versus SHR group. All data are expressed as the mean ± SD. *N* = 3 rats/group; one/two-way ANOVA followed by Tukey multiple test.

Dendritic spine maturation is an important marker of neuronal development. Recent studies have shown that dendritic and synaptic changes are more likely to be responsible for the atrophy of the hippocampus (Tata and Anderson, 2010). To determine the morphology of neurons, the Golgi-cox staining was used to investigate the dendritic complexity, number of dendritic branch and dendritic spine density in hippocampal neurons of ADHD Rat. Figure 7B showed micrographs of dendritic spines on hippocampal neuronal branches stained with Golgi-cox. Dendritic spines on dendritic segments were counted and quantified for the number of spines per 10 μm of dendritic length (Figure 7C). Quantitative analysis shown the density of dendritic spines in hippocampal DG neurons were reduced in SHR group (Figure 7C, *F* (3, 16) = 11.71, *p* < 0.0003). In contrast, the decrease of dendritic spines on DG neurons were significantly attenuated by RRP treatment (*p* < 0.05). Using Sholl analysis, we measured the alteration of dendrite branch in the hippocampus. Two-way ANOVA indicated a significant effect of distance [*F* (11, 88) = 31.49, *p* < 0.0001], treatment conditions [*F* (3, 8) = 22.18, *p* = 0.0003] and interaction [*F* (33, 88) = 2.985, *p* < 0.0001]. The intersection number in SHR DG neurons had a significantly decrease in 40 μm (*p* = 0.0033) and 60-180 μm (*p* < 0.0001) away from neuron soma as compared to the WKY. RRP treatment greatly increased the number of intersection in 100-160 μm (*p* < 0.0001) versus SHR group. But there was no significant effect on MPH treatment (*p* > 0.05).

### 3.7 RRP improved neurogenesis in the hippocampus

**Figure 8:**
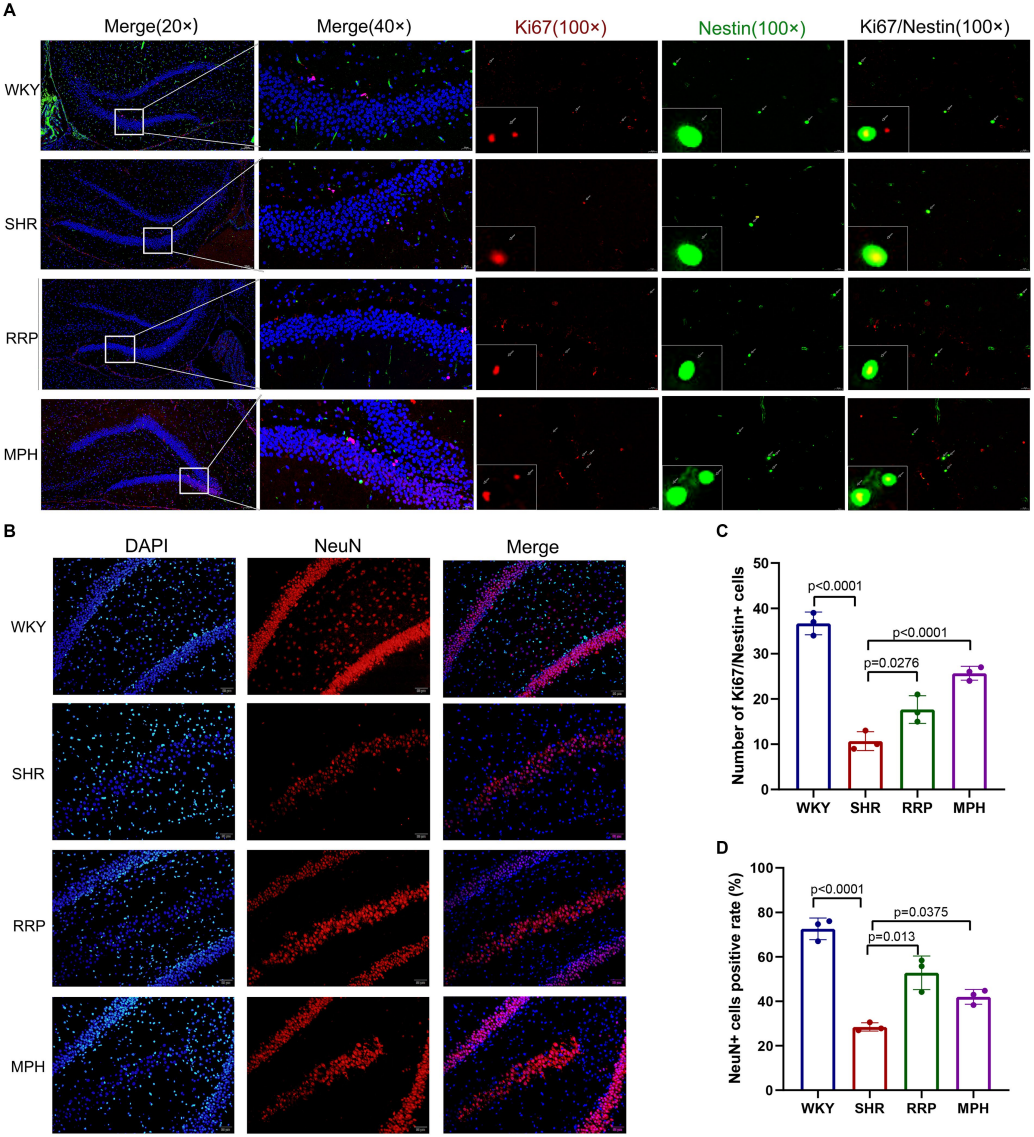
The expressions of Ki67/Nestin and NeuN using immunofluorescence staining. **(A)** Ki67/Nestin immunofluorescence staining. Nestin-positive cells (green), Ki67-positive cells(red) and DAPI-labeled nuclei (blue) are shown. Scale bar = 100/20 μm. **(B)** NeuN immunofluorescence staining. NeuN-positive cells (red) and DAPI-labeled nuclei (blue) are shown. Scale bar = 50 μm. **(C,D)** Quantitative analysis of immunofluorescence staining. All data are expressed as the mean ± SD. *N* = 3/group; one-way ANOVA followed by Tukey multiple test.

Neurogenesis refers to the formation of a large number of mature neurons, including the proliferation and differentiation of neural stem/progenitor cells into mature neurons. Ki67 is highly expressed in the nucleus of proliferating cells. And Nestin is the specific marker of neural stem cells. To evaluate the proliferation of NSC (Neural stem cell) in the hippocampus, we marked NSC with ki67 and Nestin after treatment (Figure 8A). We found that the number of Ki67 and Nestin double-labeled staining positive cells was significantly lower in SHR rats than in WKY rats (p < 0.05). Meanwhile both RRP and MPH treatment observably increased the number of total Ki67+/Nestin+ cells (Figure 8C, *F* (3, 8) = 66.99, p < 0.0001). NeuN is a soluble nuclear protein that exhibits high specificity as a marker of mature neurons. The number of positive NeuN expression in DG was used to evaluate neuronal maturation and survival. In Figure 8D [*F* (3, 8) = 80.92, *p* < 0.0001], hippocampal NeuN positivity was significantly lower in SHR than in WKY (*p* < 0.001). Compared with the SHR group, the number of NeuN-positive cells was increased in the MPH and RRP group (*p* < 0.05).

### 3.8 Effect of RRP on protein levels of Trkb, CDK5, FGF2/FGFR1 expression

**Figure 9:**
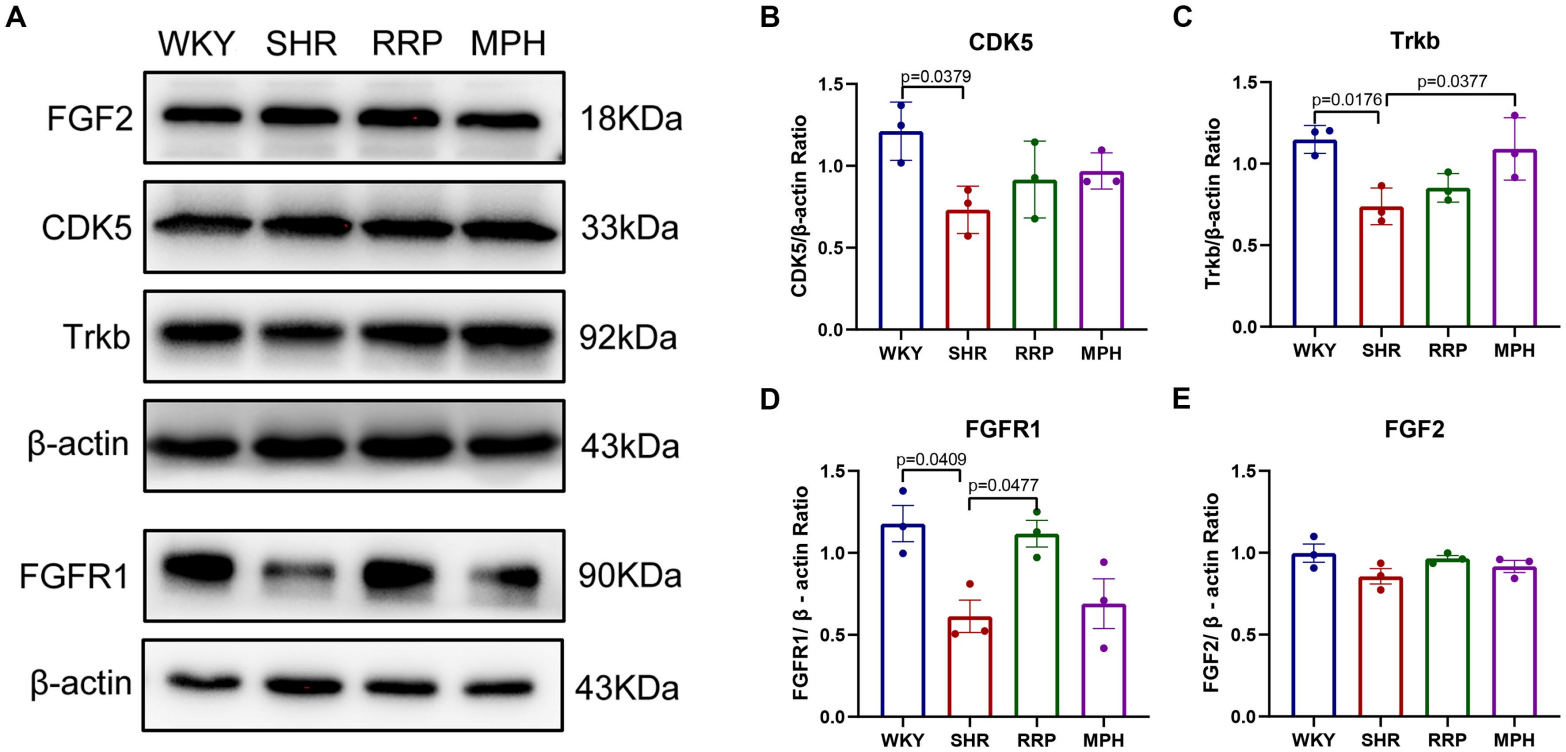
The expression of Trkb, CDK5, FGF2 and FGFR1 in hippocampus. **(A)** Protein expression of Trkb, CDK5, FGF2 and FGFR1 in hippocampus of each group by western blot. **(B–E)** Relative levels of Trkb, CDK5, FGF2 and FGFR1 (fold change relative to *β*-actin level). All data are expressed as the mean ± SD. *N* = 3 for each group; one-way ANOVA followed by Tukey multiple test.

CDK5 plays a key role as a structural cytoskeleton regulator in the brain, modulating the activity of MAPs, and also regulates neuronal maturation and migration (Cortés et al., 2019). The results of our study showed that the expression of Cdk5 protein was significantly lower in the SHR group compared to WKY group (Figure 9B, *F* (3, 8) = 3.93, *p* < 0.054). The RRP and MPH group showed increased CDK5 levels without statistically significant difference (*p* > 0.05).

Several studies suggest that BDNF/Trkb signaling pathway play a crucial role in neurodevelopment, so we assessed the effect of RRP on Trkb levels. The expression of Trkb tended to decrease in SHR rats compared to WKY rats (Figure 9C, *F* (3, 8) = 7.053, *p* < 0.0123). Compared with the SHR group, the expression of Trkb in the MPH group was significantly higher (*p* < 0.05), suggesting that MPH promotes the expression of BDNF. However, Trkb expression was not statistically different between the SHR and RRP groups.

FGFs and their receptor FGFR contribute to stem cell amplification and neurogenesis, which could make mice exhibited hyperactivity (Stevens et al., 2010). As shown in Figure 9, the FGFR1 protein expression level in the hippocampus of the SHR group was significantly decreased compared to the WKY rats (Figure 9D, *F* (3, 8) = 6.468, *p* < 0.0155), while FGF2 protein expression level was not significantly different (Figure 9E, *F* (3, 8) = 2.126, *p* < 0.1706). After drug intervention, FGFR1 protein expression levels were significantly higher in the RRP group compared to the SHR group (*p* < 0.05).

## 4 Discussion

ADHD is the most common neurodevelopmental disorder and neuroimaging shows that one of the underlying pathological mechanisms of ADHD is the abnormal development of brain regions (Grosso et al., 2023). Regarding the treatment of ADHD, more and more natural product formulations have become commonplace, with the main goal of avoiding or reducing the use of psychotropic drugs. Therefore, based on the pathogenesis of ADHD and the pharmacological functions of RRP, we hypothesized that RRP may be a safe and effective treatment for ADHD. Though detecting the UHPLC–MS characteristics of RRP extract, we identified several potential compounds containing catalpol, acteoside, and echinacoside. In particular, catalpol has been shown to have good neuroprotective effects in the treatment of a variety of neurological disorders, including Alzheimer’s disease (Zhang et al., 2021; Du et al., 2022), Parkinson’s disease (Wang et al., 2019), neuropsychiatric disorders (Wang Y. L. et al., 2021; Wang J. et al., 2021), and stroke (Sun et al., 2023). In our previous study, RRP and catalpol could improve the energy metabolism disorder and exerts neuroprotective role in prefrontal cortex of SHR (Yuan et al., 2018, 2019).

In this study, we used behavioral test, brain tissue staining and Western blot analysis to evaluate behavior and hippocampal neurogenesis in SHR rats. In this study, the results of EPM test showed that both MPH and RRP could reduce the number of entries into the open arm and the residence time of the open arm in SHR rats, and effectively control the impulsive behavior. The results of OFT suggested that RRP exerted its effects later than MPH, but over time, RRP may be more effective than MPH in controlling spontaneous activity. The results of the MWM test showed that the escape latencies of the SHR, MPH and RRP groups decreased with training time, and were significantly lower than those of WKY group at the same time. Interestingly, we found that SHR had a shorter escape latency than WKY and performed better in MWM. The probe trial suggested that the MPH and RRP could improve the spatial learning and memory ability of the SHR rats. In conclusion, RRP could alleviate ADHD-like behavioral characteristics of young SHR rats, that is, reduced their spontaneous activity and impulsive behavior, and improved learning and memory function. In addition, WKY rats, as the control group, had prolonged latency due to their prolonged swimming stagnation, and the number of crossing platforms and residence time in the target quadrant were abnormally reduced. The inevitable significant differences caused by this result made it controversial whether WKY rats could be used as the best control for SHR rats. So how to optimize the selection of the control group for SHR rats is worthy of further study.

Perinatal exposure could lead to ADHD-like hyperactivity and impulsivity, and loss of dentate gyrus neurons in male offspring (Xi et al., 2022). Previous studies have shown that 20–25% of neurons may be dead or severely dysfunctional in children with ADHD (Derbyshire and Maes, 2023). Accumulating evidence suggests that neuronal loss and synaptic damage are major drivers of impaired hippocampal dependent learning and memory. Therefore, the structural and functional abnormalities in hippocampal structures already observed in neurodevelopmental disorders were strongly associated with ADHD behavior. To investigate the potential neuroprotective effect of RRP against hippocampal apoptosis in SHR, Nissl and TUNEL staining was performed. The results of staining indicated that RRP had the effect of attenuating the neuronal loss in SHR rats. To investigate the effect of RRP on synaptic plasticity, we calculated the density and length of dendritic spines by Golgi-Cox staining and tracked the complexity of neuronal dendrites using Sholl analysis. We found that treatment with RRP could promote hippocampal dendritic complexity and dendritic spine density in SHR rats.

In addition, we used transmission electron microscopy to observe the morphology of neurons. Previous studies have shown that abnormal synaptic ultrastructure dysfunction may be involved in the pathogenesis of ADHD (Bai et al., 2022), and MPH can improve the synaptic ultrastructure of the prefrontal cortex in SHR rats. In our study, we found that the myelin structure of SHR rat neurons was sparse, and the microfilament microtubule structure in many axons was disappeared. This suggested that SHR rats have impaired neuronal myelin development, which may be involved in the development of ADHD. Neural stem cells (NSCs) can self-renew and provide new neurons to the brain tissue (Chang et al., 2024). Thus, endogenous NSCs could potentially be used for brain repair. The proliferation and survival of NSCs are key factors in the regulation of neurogenesis. Therefore, we examined Ki67 and Nestin, which represent stem cells division, to understand the proliferation of hippocampal NSCS. We found that RRP could significantly increase the number of hippocampal stem cells. Neural stem cells/progenitor cells (Nestin) develop into intermediate progenitor cells that express doublecortin (DCX) in 2 weeks and further develop into mature neurons (NeuN) in 3 weeks (Sierra et al., 2010). Therefore, we can determine whether RRP could promote the expression of mature neurons by detecting the expression of NeuN-positive cells. Our study demonstrated that RRP administration not only increased the number of hippocampal neurons at different stages, such as neural stem cells and mature neurons, but also improved their function, such as increasing the number of dendritic branches and dendritic spines and promoting synaptic ultrastructural integrity. It was shown that RRP treatment accelerated neuronal proliferation, maturation and survival and promoted the functional maturation of hippocampal neural networks.

TrkB is a high-affinity receptor for BDNF, and both are widely distributed in the cortex, striatum and hippocampus. As a signaling pathway ligand, BDNF binds specifically to its receptor TrkB and directs the autophosphorylation of TrkB, thereby activating the BDNF/TrkB signaling pathway and exerting the neuron-protective effect (Yamada and Nabeshima, 2003; Yin et al., 2018). In the present study, we found that compared with WKY rats, the expression level of hippocampal TrkB protein was significantly reduced in SHR rats, and MPH was able to significantly increase the expression level of TrkB protein in SHR rats, which was in agreement with the previous reports. In addition, it has been shown that TrkB plays an important role in the formation of neuronal myelin lamellar structure. Combined with the results of myelin ultrastructure under electron microscope in the present experiments, the increase in the expression of TrkB may be the mechanism that RRP promotes the structural integrity of myelin and development in the neurons of SHR rats. Cdk5 is a cyclin-dependent kinase family member (Shah and Lahiri, 2017). Cdk5 plays a key role in neuronal development by regulating neuronal migration, neurite growth, axon guidance and synapse formation (McLinden et al., 2012). Previous studies have shown that aberrant Cdk5 expression during development may contribute to the delayed or atypical brain maturation observed in ADHD subjects, and thus may influence the etiology of ADHD. The result in this study revealed that the expression of Cdk5 protein was significantly lower in the SHR group compared to WKY group. The RRP and MPH group showed increased CDK5 levels without statistically significant difference. Therefore, the hypothesis that RRP may be involved in the etiology of ADHD by modulating the Cdk5 signaling pathway needs to be further tested.

FGFs consist of a considerable family of secreted peptides that control a variety of neurodevelopmental processes such as cell division, proliferation, differentiation and migration (Zhang et al., 2023). Fibroblast growth factor receptor 1 (FGFR1) is one of the receptors for FGF, and FGFR1 is required for hippocampal growth because it promotes the proliferation of hippocampal progenitor and stem cells during development (Ohkubo et al., 2004). In animal models with disrupted FGFR1 dependent signaling, studies have found changes in anxiety-like behavior, altered stress response, and social interaction (Stevens et al., 2023). FGF2, also known as basic fibroblast growth factor, is widely distributed in the central nervous system. It has been shown that FGF2 is involved in promoting neurogenesis, inhibiting neurotoxicity and extended neurons life-span (Tyebji and Hannan, 2017; Ilieva et al., 2019). In this study, we found that FGF2 protein expression in SHR rats was not significantly different from that in WKY rats, but FGFR1 protein expression was significantly lower than that in WKY rats. MPH had no significant effect on FGFR1 protein expression; whereas RRP had a significant up-regulation of FGFR1 expression in SHR rats. In summary, it appeared that dysregulation of FGFRs, rather than FGFs, was associated with ADHD. Compared with MPH, RRP showed stable regulation of FGFR.

This study has several limitations. With the complex signaling network interactions, we only demonstrate that FGFRs may be one of the potential mechanistic pathways affected by RRP treatment. Therefore, additional work is needed to evaluate the effects of RRP and MPH on the other regulatory proteins involved in neurodevelopment. Due to the abundance of chemicals in the RRP formulation, our study can only provide very limited insights into the promising active compounds that contribute to its neurodevelopmental effect. Therefore, we should further explore the potential mechanisms and active compounds of RRP’s anti-ADHD behavior and neurogenic effects to provide solid experimental evidence for future clinical trials. We found some alterations in MWM behavioral test in WKY, so how to optimize the selection of the control group for SHR rats is worthy of further study. In addition, the result of TUNEL staining has an unavoidable limitation that cannot show specific neuronal pattern due to our incomplete consideration of experimental design. More work should be done to prove the effect of RRP on neuronal apoptosis. Finally, we studied only male rats to demonstrate the potential effects of RRP on behavior and neurodevelopment. Further experiments are needed to assess sex-related differences.

In conclusion, our study demonstrated the presence of ADHD-like behavioral characteristics and hippocampal neuronal developmental deficits in SHR rats and revealed key proteins that may be involved in this mechanism. The present study provides a novel perspective on the protective effects of RRP against neurodevelopmental deficits in ADHD and reveals potential new therapeutic targets for ADHD.

## Data availability statement

The original contributions presented in the study are included in the article/supplementary material, further inquiries can be directed to the corresponding authors.

## Ethics statement

The animal study was approved by the Animals Ethics Committee of China Science and Technology Industry Holding (Shenzhen). The study was conducted in accordance with the local legislation and institutional requirements.

## Author contributions

RS: Data curation, Formal analysis, Supervision, Writing – original draft, Writing – review & editing. HY: Investigation, Methodology, Supervision, Writing – original draft. JW: Investigation, Methodology, Supervision, Writing – review & editing. KZ: Investigation, Supervision, Writing – review & editing. YX: Investigation, Methodology, Writing – review & editing. YZ: Supervision, Validation, Writing – review & editing. XN: Funding acquisition, Methodology, Project administration, Resources, Validation, Writing – review & editing. MH: Project administration, Supervision, Validation, Writing – review & editing.
